# Not all stressors are equal: behavioral and endocrine evidence for development of contextual fear conditioning after a single session of footshocks but not of immobilization

**DOI:** 10.3389/fnbeh.2012.00069

**Published:** 2012-10-29

**Authors:** Núria Daviu, Raúl Delgado-Morales, Roser Nadal, Antonio Armario

**Affiliations:** ^1^Institut de Neurociències, Universitat Autònoma de BarcelonaBellaterra, Barcelona, Spain; ^2^Red de trastornos adictivos (RTA), Instituto de Salud Carlos IIIMadrid, Spain; ^3^Unitat de Fisiologia Animal (Facultat de Biociències), Universitat Autònoma de BarcelonaBellaterra, Barcelona, Spain; ^4^Unitat de Psicobiologia (Facultat de Psicologia), Universitat Autònoma de BarcelonaBellaterra, Barcelona, Spain

**Keywords:** contextual fear conditioning, hypothalamic-pituitary-adrenal axis, stress, footshock, immobilization, freezing

## Abstract

Exposure of animals to footshocks (FS) in absence of any specific cue results in the development of fear to the compartment where shocks were given (contextual fear conditioning), and this is usually evaluated by time spent freezing. However, the extent to which contextual fear conditioning always develops when animals are exposed to other stressors is not known. In the present work we firstly demonstrated, using freezing, that exposure of adult rats to a single session of FS resulted in short-term and long-term contextual fear conditioning (freezing) that was paralleled by increased hypothalamic-pituitary-adrenal (HPA) activation. In contrast, using a similar design, no HPA or behavioral evidence for such conditioning was found after exposure to immobilization on boards (IMO), despite this stressor being of similar severity as FS on the basis of standard physiological measures of stress, including HPA activation. In a final experiment we directly compared the exposure to the two stressors in the same type of context and tested for the development of conditioning to the context and to a specific cue for IMO (the board). We observed the expected high levels of freezing and the conditioned HPA activation after FS, but not after IMO, regardless of the presence of the board during testing. Therefore, it can be concluded that development of fear conditioning to context or particular cues, as evaluated by either behavioral or endocrine measures, appears to be dependent on the nature of the aversive stimuli, likely to be related to biologically preparedness to establish specific associations.

## Introduction

When rats or mice are exposed to an aversive stimulus such as footshocks (FS) in a specific compartment, the animals develop fear to the place where shocks were given and this has been termed contextual fear conditioning. If additional cues that predict shocks are given (tone or light), the animals also develop cue-fear conditioning, but still maintain a certain level of contextual fear conditioning (i.e., Maren et al., 1994). By classical (pavlovian) conditioning, pairing of an initially neutral stimulus (a particular environment or specific cues) with an aversive (unconditioned stimulus, US) resulted in the development of a conditioned response (CR) to the mere presentation of the previously neutral stimulus (conditioned stimuli, CS). Cue and contextual fear conditioning has been for decades the typical paradigm for the study of factors and neurobiological mechanisms involved in aversive learning processes. Cue and context fear conditioning can develop, under appropriate conditions, even after a single shock (i.e., Fanselow, 1980; Rudy, 1993), thus demonstrating a strong biological predisposition to this type of learning. Ample evidence has been obtained for a critical role of stress-induced glucocorticoid release in the development of emotional memory and shock-induced fear conditioning (Sandi and Pinelo-Nava, 2007). More specifically, it appears that activation of glucocorticoid receptors (GR) in the basolateral nucleus of the amygdala is critical for β-adrenergic receptors in this area to potentiate emotional memory (de Quervain et al., 2009).

FS-induced fear conditioning is usually evaluated by time spent freezing after exposure to the CS. However, there is evidence that exposure to the CS can also elicit, depending on the experimental conditions, other types of behaviors, including avoidance, hypo-activity, risk assessment, suppression of ongoing operant behavior or fear-potentiated startle response (i.e., Davis, 1990; Radulovic et al., 1998; Antoniadis and McDonald, 1999; Laxmi et al., 2003). In addition to behavior, physiological parameters such as plasma levels of hypothalamic-pituitary-adrenal (HPA) hormones can also be useful as makers of fear conditioning (see Armario et al., 2012 for review).

In addition to their paramount importance in the study of emotional learning, procedures involving FS are also widely used in animal models for psychopathology, on the assumption that excessive response to shocks and corresponding enduring memory about the situation can give us some clues about the bases for the development of pathological anxiety, including post-traumatic stress disorder (PTSD) (Rau et al., 2005). In fact, it has been observed that under certain conditions, exposure to FS also resulted in long-lasting alterations of behavior in novel environments. More specifically, shock-exposed animals develop hypo-activity in environments not previously associated to shock (i.e., van Dijken et al., 1992a,b; Van den Berg et al., 1998), although this is difficult to interpret in terms of enhanced anxiety-like behavior (Radulovic et al., 1998; Kamprath and Wotjak, 2004; Daviu et al., 2010). Interestingly, hypo-activity in novel environments appears to be associated to the development of shock-induced contextual fear conditioning (Radulovic et al., 1998; Daviu et al., 2010), suggesting some kind of generalization of fear/anxiety to environments completely unrelated to that in which the animals received the shocks.

In recent years, another stress paradigm, exposure to cat odor, has been developed that also results in contextual/cue (a cloth or piece impregnated with the odor) fear conditioning. This is reflected in behavioral inhibition, immobility, and avoidance of the odor-associated cue (Dielenberg et al., 1999, 2001; McGregor and Dielenberg, 1999; Blanchard et al., 2001, 2003; Takahashi et al., 2008; Muñoz-Abellán et al., 2009), together with activation of the HPA axis and the sympathetic system (Dielenberg et al., 2001; Muñoz-Abellán et al., 2009), when animals are again exposed days later to the same context of odor exposure. Interestingly, a single exposure of rats or mice to a cat (predator) or to cat's odors is able to induce long-lasting increases in anxiety-like behavior, as evaluated by the elevated-plus maze (EPM) and the acoustic startle response (ASR) (i.e., Adamec and Shallow, 1993; Cohen et al., 2003; Muñoz-Abellán et al., 2008).

The above data indicate that FS and predator odor are able to induce long-lasting contextual fear conditioning, but also long-lasting changes in behavior of animals in novel environments. In contrast, exposure to two stressors considered of high intensity on the basis of the physiological changes they elicit, immobilization on boards (IMO) or a prolonged session of inescapable electric tail-shocks typical of the learned helplessness paradigm (IS-LH) (Maier et al., 1986; Fleshner et al., 1995; Vallès et al., 2000; Márquez et al., 2002), has been found to induce important behavioral changes for a few days after the stressors, but most changes apparently vanished after 1 week (i.e., Maier, 1984; Reinstein et al., 1984; Belda et al., 2008). We have hypothesized that failure to find long-lasting changes in anxiety after exposure to these two severe stressors may be, at least in part, related to difficulties for animals to establish contextual fear conditioning to these particular stressors (Armario et al., 2008). However, to our knowledge, there is no report assessing whether or not contextual fear conditioning has developed with these two stressors. In fact, considering the absence of studies on fear conditioning with stressors other than FS or cat odor, it is unclear whether or not development of contextual fear conditioning is not a general property of aversive stimuli but a particular property of a restricted set of stressors. Thus, in the present work we studied in adult male rats possible differences between exposure to FS or IMO in a particular environment regarding the development of contextual fear conditioning. Our results indicate that contextual fear conditioning develops only after exposure to FS but not IMO, demonstrating that development of contextual fear conditioning in rats is not a universal property of all aversive stimuli that activate the HPA axis.

## Materials and methods

### Animals and general procedure

Two month old male Sprague–Dawley rats (average body weight 288 ± 30 g) were used. These rats were obtained from the breeding center of the Universitat Autònoma de Barcelona. The animals were housed in pairs in 1000 cm^3^ plastic cages, under standard conditions of temperature (22 ± 1°C) and maintained on a 12 h light −12 h dark schedule (lights on at 08:00 h), with *ad libitum* access to food (SAFE-diet A04, Panlab, Barcelona, Spain) and water. The animals were allowed to acclimatize to the housing conditions for at least 1 week before the beginning of the experimental treatments, which were carried out in the morning. The experimental protocol was approved by the Committee of Ethics of the Universitat Autònoma de Barcelona, followed the “Principles of laboratory animal care” and was carried out in accordance with the European Communities Council Directives (86/609/EEC).

Animals were handled for three consecutive days (approximately 2 min a day) and the last day of handling, they were subjected to tail-nick procedure to habituate animals to this blood sampling procedure. The tail-nick consisted of gently wrapping the animals with a cloth, making a 2 mm incision at the end of one of the tail veins and then massaging the tail while collecting, within 2 min, 300 μl of blood into ice-cold EDTA capillary tubes (Sarsted, Granollers, Spain). After centrifugation at 4°C, plasma was stored at −20°C. In all experiments, cage-mates were processed simultaneously, including blood sampling (two experimenters were sampling at the same time and a third was gently holding the two rats). Tail-nick procedure is extensively used in our lab because low resting levels of hormones are obtained (i.e., Belda et al., 2004; Vahl et al., 2005). Animals were always stressed in a room different from the animal room and blood sampling room.

The stressors used were FS or IMO. Scramble shocks 1.5 mA, 3 s of duration were administered every 60 s. IMO rats were stressed by taping their four limbs to metal mounts attached to a board (García et al., 2000). Head movements were restricted with two plastic pieces (7 × 6 cm) placed in each side of the head and the body was subjected to the board by means of a piece of plastic cloth (10 cm wide) attached with *Velcro* that surrounded all the trunk.

### Apparatuses and behavioral recording

The small shock chambers of Experiment 1 were clear Plexiglas boxes (19.7 × 11.8 × 20.0 cm) with a metal removable grid floor of 15 stainless steel rods, 0.4 cm diameter and spaced 0.9 cm centre to centre (Cibertec, Madrid, Spain). The open-fields (OF) of the Experiment 2 were gray rectangular plastic (56 × 36.5 × 31 cm) boxes. The large shock chambers of Experiment 3 were clear Plexiglas boxes (57 × 41 × 70 cm) with a metal removable grid floor of 44 stainless steel rods, 0.4 cm diameter, spaced 1.5 cm center to center (Panlab S.L.U, Barcelona). The apparatuses were always cleaned carefully between animals with tap water solution containing ethanol (5%, v/v).

In Experiment 1, video cameras (Sony SSC-M388 CE, BW) were placed in front of the FS chamber and recorded the two cage-mate animals simultaneously. In Experiment 2, cameras were suspended from the ceiling (1.20 m above the surface of the OF) and two OF were recorded simultaneously. Activity of the animals was evaluated in 5 min blocks by video-tracking using the center of gravity of the animal (Smart version 2.5.19, Panlab S.L.U, Barcelona). In Experiment 3, one camera was placed in front of the chamber to assess freezing behavior (by a stop-watch), and another camera was suspended from the ceiling to evaluate motor activity by video-tracking. The images were transferred to a JVC VR-716 digital video recorder. The video recorder sampled the position of the rat (8.3 samples/s) and was used to transfer the videos to a computer for subsequent analysis. An experimenter blind to the treatment measured activity or freezing. The latter behavior involves the absence of all movement, except for respiratory-related movements.

### Biochemical assays

Plasma ACTH and corticosterone levels were determined by double-antibody radioimmunoassay (RIA). In brief, ACTH RIA used ^125^I-ACTH (PerkinElmer Life Science, Boston, USA) as the tracer, rat synthetic ACTH 1–39 (Sigma, Barcelona, Spain) as the standard and an antibody raised against rat ACTH (rb7) kindly provided by Dr. W. C. Engeland (Department of Surgery, University of Minnesota, Minneapolis, USA). The characteristics of the antibody have been described previously (Engeland et al., 1989) and we followed a non-equilibrium procedure. Corticosterone RIA used ^125^I-corticosterone-carboximethyloxime-tyrosine-methylester (ICN-Biolink 2000, Barcelona, Spain), synthetic corticosterone (Sigma, Barcelona, Spain) as the standard and an antibody raised in rabbits against corticosterone–carboximethyloxime-BSA kindly provided by Dr. G. Makara (Inst. Exp. Med., Budapest, Hungary). The characteristics of the antibody and the basic RIA procedure have been described previously (Zelena et al., 2003). All samples to be statistically compared were run in the same assay to avoid inter-assay variability. The intra-assay coefficient of variation was 3.8 % for ACTH and 7.8 % for corticosterone. The sensitivity of the assays was 12.5 pg/ml for ACTH and 1 ng/ml for corticosterone.

### Statistical analysis

Data were analyzed by the Statistical Program for Social Sciences (SPSS), version 17. Behavioral and hormonal response at a single-point was analyzed by means of a generalized linear model (GENLIN) (McCulloch and Searle, 2001). A generalized estimated equation model (GEE) was used to analyze repeated measures data (Hardin and Hilbe, 2003). The within and between-subjects factors used are indicated in each experiment. GENLIN and GEE models are more flexible statistical tool than the general linear model for the following reasons: (1) you can choose between several types of distribution of your data (normal, binomial, Poisson, gamma, or inverse-Gaussian), (2) you can run the analysis even if you have some missing data in your repeated measures data, (3) you do not need homogeneity of variance. In all cases, if a statistical significant interaction was found, additional pair-wise comparisons (Bonferroni sequential adjustment) were made. As a method of estimation, the maximum likelihood (ML) was used. Normality distribution and identity as a link function was always used. The significance of the effects was determined by the Wald chi-square statistic.

## Experimental designs and results

### Experiment 1

The objective of the experiment was to demonstrate that contextual fear conditioning immediately after the FS session may be reflected in both behavioral (freezing) and endocrine changes and that such conditioning would be maintained for days after FS.

#### Design

**Figure 1:**
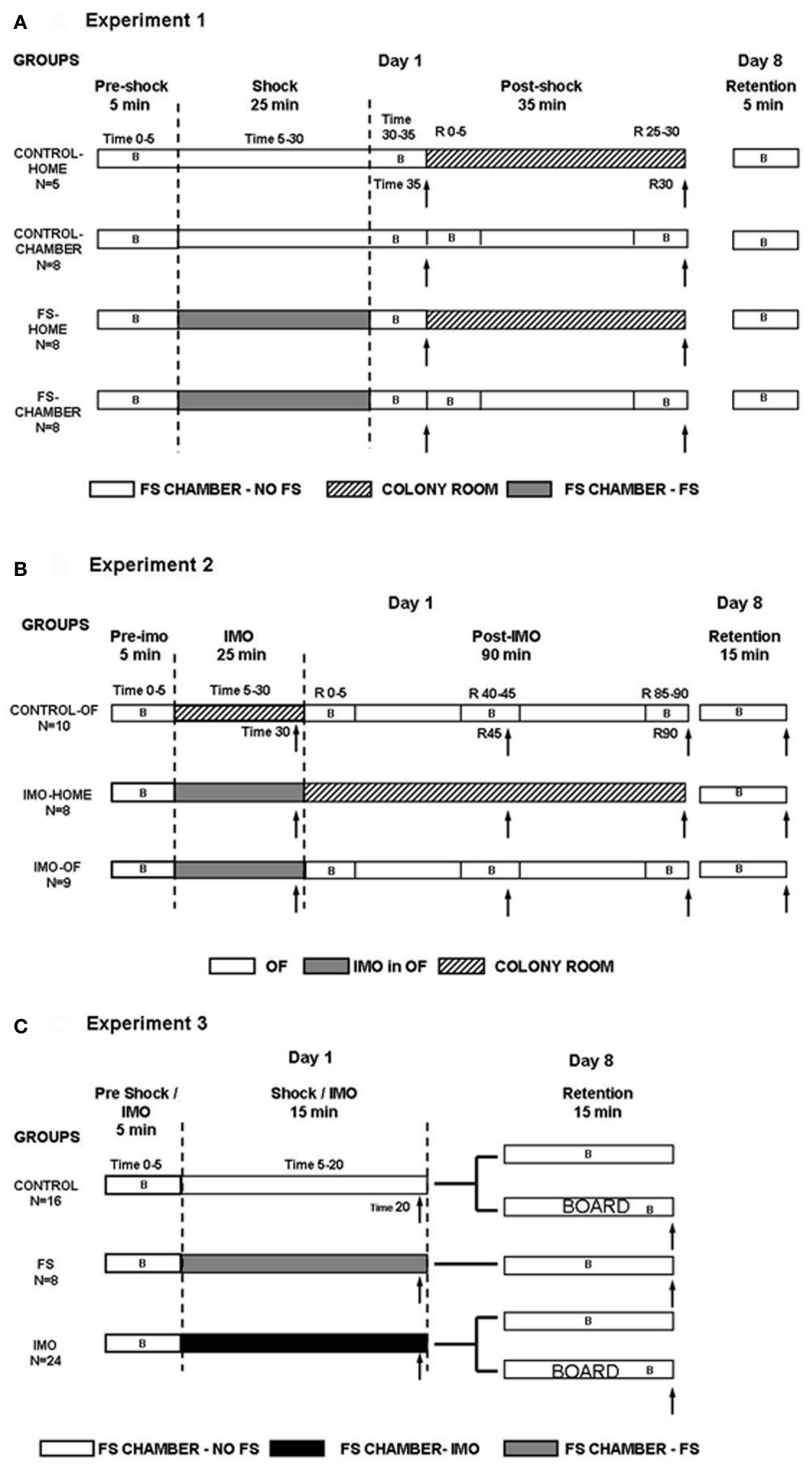
**Summary of the experimental design for Experiment 1 (A), Experiment 2 (B), and Experiment 3 (C).** B: periods of behavioral assessment. ↑, blood sampling.

On day 1, all animals were initially exposed for 5 min to small shock chambers without receiving FS (pre-shock time 0–5). Then, treatments differed in function of the experimental group (see detailed procedure in Figure 1A): (1) control-home rats (*n* = 5) were additionally maintained for 30 min in the FS chamber without FS; then, they were blood sampled (time 35) and moved to their regular home-cages in the animal room, being sampled again after an additional 30 min period (time R30); (2) control-chamber rats (*n* = 8) were additionally exposed for 30 min to the FS-chamber without FS, immediately sampled (time 35) and returned to the FS chamber for an additional 30 min period, after which they were sampled again (time R30); (3) FS-home rats (*n* = 8) were exposed for 25 min to 25 shocks in total (shock time 5–30), maintained for 5 min without shocks (post-shock time 30–35), sampled (time 35 min) and moved to their regular home-cages at the animal room for an additional 30 min period followed by sampling (time R30); and (4) FS-chamber rats (*n* = 8) were exposed for 25 min to FS (shock time 5–30), maintained for 5 min without shocks, sampled (post-shock time 35) and returned to the FS chamber for an additional 30 min shock-free period followed by sampling (post-shock time R30). The rationale to have “home” and “chamber” rats was to study whether rats that remained in the chamber after the shocks showed a slow hormonal recovery as a physiological measure of contextual fear conditioning.

Behavior was recorded in the shock chamber for all groups during the 5 min pre-shock period (time 0–5) and for the control-chamber and FS chamber groups at the following times: post-shock time 30–35, immediately after sampling (time R 0–5), and during the last 5 min in the FS chamber, just before the second sampling (time R 25–30). On day 8 (retention), all rats were again exposed to the FS chamber for 5 min (without FS), to evaluate freezing as a measure of contextual fear conditioning.

The statistical analysis included two between-subjects factors: (1) SHOCK (control and shocked) and (2) POST-SHOCK CONDITION (home and chamber). When repeated measures were included in the analysis, the within-subjects factors were SAMPLING TIME (2 levels) for endocrine data or BLOCK (3 levels) for freezing data.

#### Results

**Figure 2:**
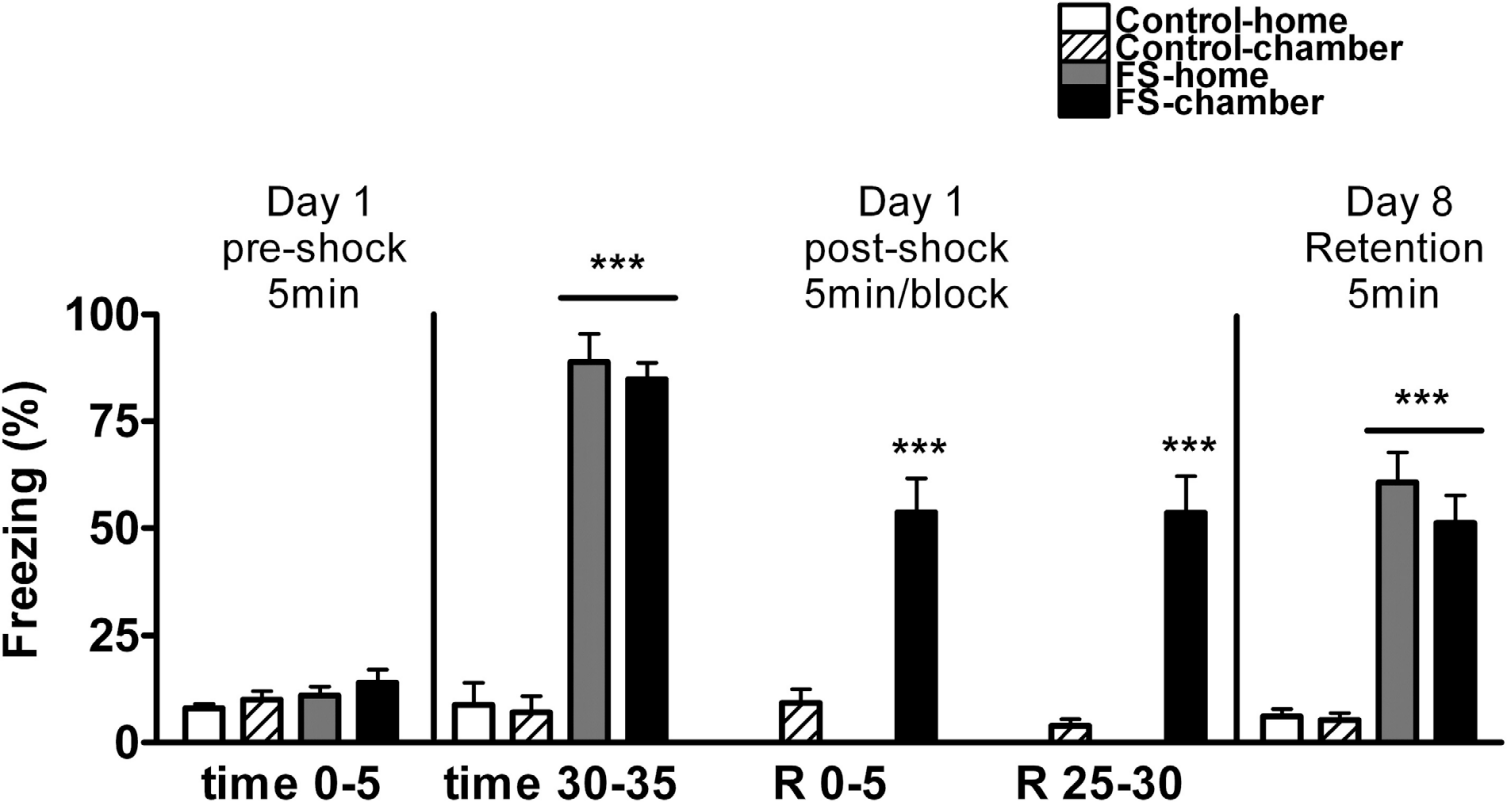
**Percentage of time spent freezing for Experiment 1 on day 1 (pre-shock period and several post-shock periods), and day 8 (retention).** Means and SEM are represented. The groups were as follows: control-home, not shocked during the 35 min exposure to the shock chamber and returned to the home-cage after that; control-chamber, not shocked during the 35 min in the chamber and maintained in the shock chamber for an additional period of 30 min (R 0–30); FS-home, shocked in the chamber and returned to their home-cages after that; FS chamber, shocked in the chamber and maintained in the shock chamber for an additional period of 30 min without shocks. ^***^p < 0.001 vs. control groups.

We first analyzed whether or not baseline freezing differed among the groups before FS (pre-shock time 0–5) and this analysis, revealed no group differences (Figure 2). Then, we analyzed whether post-shock freezing (time 30–35) differed in function of the experimental group. The analysis revealed, as expected, a significant effect of shock (Wald *X*^2^ (1) = 366.65, *p* < 0.001), but not of post-shock condition, thus demonstrating that the two shocked groups were homogeneous (Figure 2). After that, freezing behavior of control-chamber and FS-chamber groups was compared over time. Statistical analysis revealed significant effects of shock (Wald *X*^2^ (1) = 148.04, *p* < 0.001), block (Wald *X*^2^ (2) = 20.47, *p* < 0.001) and the interaction block *X* time (Wald *X*^2^ (2) = 19.16, *p* < 0.001). Further analysis demonstrated high levels of freezing in FS group as compared to control group (*p* < 0.001), although a moderate decrease of freezing over time was observed in the two groups, but particularly in the FS group.

When exposed again to the FS chamber on day 8 for retention (Figure 2), the analysis of freezing behavior revealed a significant effect of shock (Wald *X*^2^ (1) = 120.12, *p* < 0.001), but not post-shock condition, reflecting that levels of freezing were independent of whether or not, on day 1, the animals returned to their home-cages immediately after shocks.

**Figure 3:**
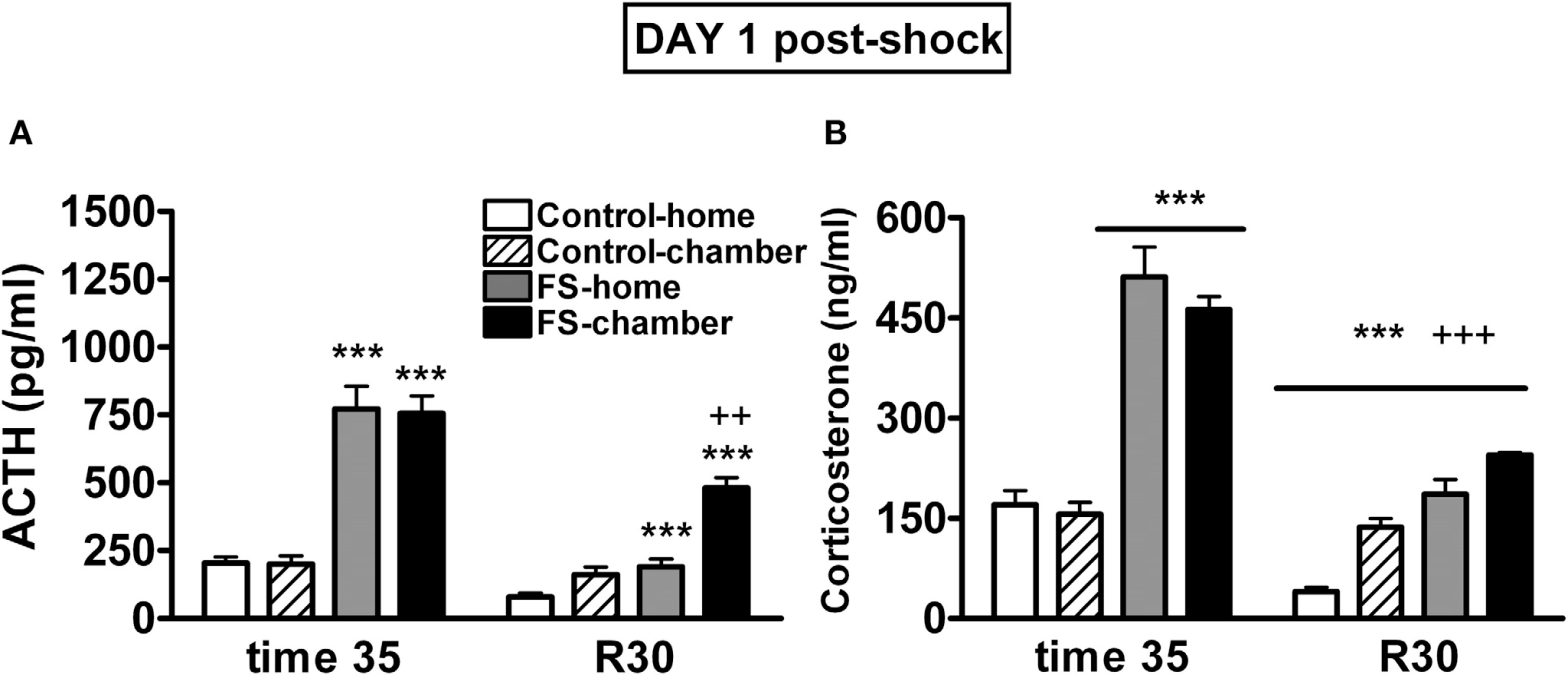
**Plasma ACTH (A) and corticosterone (B) levels on day 1 for Experiment 1.** Means and SEM are represented. The groups were as follows: control-home, not shocked during the 35 min exposure to the shock chamber and returned to the home-cage after that; control-chamber, not shocked during the 35 min in the chamber and maintained in the shock chamber for an additional period of 30 min (R 0–30); FS-home, shocked in the chamber and returned to their home-cages after that; FS-chamber, shocked in the chamber and maintained in the shock chamber for an additional period of 30 min without shocks. Blood sampling was done in the post-shock period at times 35 and at R30. ^***^p < 0.001 vs. control groups; ^++^, ^+++^: *p* < 0.01 and *p* < 0.001 vs. corresponding home groups.

Plasma ACTH levels on day 1 were analyzed using shock and post-shock conditions as the between-subjects factors and sampling time as the within-subjects factor. As can be seen in Figure 3A, the analysis revealed significant effects of shock (Wald *X*^2^ (1) = 126.49, *p* < 0.001), post-shock condition (Wald *X*^2^ (1) = 6.52, *p* < 0.05), sampling time (Wald *X*^2^ (1) = 126.95, *p* < 0.001) and the interactions shock *X* sampling time (Wald *X*^2^ (1) = 58.45, *p* < 0.001), post-shock condition *X* sampling time (Wald *X*^2^ (1) = 19.00, *p* < 0.001), and the second order interaction shock *X* post-shock condition *X* sampling time (Wald *X*^2^ (1) = 5.918, *p* < 0.05). Further analysis indicated that immediately after shocks, the two shocked groups showed high levels of ACTH as compared to the respective non-shocked groups (*p* < 0.001 in both cases), with no differences among the groups in function of the post-shock condition. During the post-shock period (R30), the FS-home group showed higher levels than the control-home group (*p* < 0.001) and the highest levels were observed in the FS-chamber group that differed significantly from control-chamber (*p* < 0.001) and from FS-home group (*p* < 0.01). That is, during the post-shock period, the FS rats that were returned to their home-cages showed higher levels of ACTH than control-home rats, but ACTH levels were even higher in those FS rats maintained in the chamber after the shocks.

The analysis of plasma corticosterone levels on day 1 (Figure 3B) revealed significant effects of shock (Wald *X*^2^ (1) = 178.74, *p* < 0.001), sampling time (Wald *X*^2^ (1) = 235.62, *p* < 0.001) and the interactions shock *X* sampling time (Wald *X*^2^ (1) = 76.60, *p* < 0.001) and post-shock condition *X* sampling time (Wald *X*^2^ (1) = 23.32, *p* < 0.001). The decomposition of the interactions indicated that immediately after shocks, the two FS groups showed high levels of corticosterone as compared to the non-shocked groups (*p* < 0.001). Moreover, at R30, significant effects of shock and post-shock conditions were found (*p* < 0.001 in the two cases), reflecting that animals previously exposed to shock showed higher corticosterone levels than non-shocked animals and that those maintained in the chamber showed higher levels than those that were returned to their home-cages.

In Experiment 1, both FS groups showed a much higher activation of the HPA axis than controls as a consequence of FS. Interestingly, although plasma ACTH and corticosterone levels decreased in the two FS groups during the post-shock period, the FS-chamber group showed higher levels of ACTH than the FS-home group, demonstrating that maintenance of FS animals in the FS chambers not only induced freezing, but also resulted in a more sustained activation of the HPA axis as compared to FS-home group. When animals are exposed seven days later to the FS chamber, both FS groups showed high levels of freezing as compared to non-shocked groups, therefore, the longer exposure to the context on day 1 had no effect on contextual fear conditioning (measured by freezing).

### Experiment 2

The previous experiment demonstrated that rats exposed to shocks showed clear evidence for contextual fear conditioning. We decided to use an analogous design with IMO as the stressor, although some changes were introduced in the protocol considering the specific characteristics of IMO and the expected results. First, we did not include the control-home group due to the low hormonal response observed in Experiment 1. Second, an OF, much larger in size than the small shock chambers of Experiment 1, was chosen as the small chambers were too small to accommodate the IMO boards. Third, due to the larger size of the OF, which may affect the expression of fear conditioning, we measured both hypo-activity and freezing a putative measures of fear conditioning (i.e., Radulovic et al., 1998; Laxmi et al., 2003). Moreover, to further corroborate behavioral measures, we also evaluated HPA function as a reflection of conditioning. This was based on our positive results in the preceding experiments with short-term conditioning and results from the literature that have demonstrated that HPA activation reflects fear conditioning (Van de Kar et al., 1991; Campeau et al., 1997; Merino et al., 2000; Muñoz-Abellán et al., 2009; Daviu et al., 2010). Finally, a prolonged period of exposure to the OF during fear conditioning testing was chosen (15 min) because, in our hands, HPA activation consistently reflects fear conditioning when exposure to the context lasted for 15 min instead of the 5 min exposure typically used when only freezing is evaluated (Muñoz-Abellán et al., 2009; Daviu et al., 2010; Armario et al., 2012).

#### Design

On day 1, all rats were initially exposed for 5 min to the OF (pre-IMO time 0–5). After that, the treatment differed in function of the particular experimental group (Figure 1B): (1) control-OF (*n* = 10) rats were returned to their regular cages in the animal room for 25 min (time 5–30), then sampled (time 30) and exposed again to the OF for 90 min; during this latter period rats were additionally sampled at 45 and 90 min (times R45 and R90); (2) IMO-home (*n* = 8) rats were immobilized for 25 min (IMO time 5–30) within the OF, then sampled (time 30) and returned to their regular cages in the animal room, being sampled again in the post-IMO period at R45 and R90; and (3) IMO-OF (*n* = 9) rats were immobilized for 25 min (time 5–30) within the OF; then, sampled (time 30) and returned again to the OF for 90 min, with additional sampling in the post-IMO period at R45 and R90 min. Control-OF rats were not maintained in the OF during the initial 25 min period when the other groups were exposed to IMO because prolonged exposure to the OF may progressively reduce activity/exploration as a consequence of habituation, whereas this was unlikely in IMO groups as the rats had not opportunity to explore the OF while immobilized. Behavior was recorded as follows: (1) in the three groups during the first 5 min in the OF (pre-IMO time 0–5); (2) in the control-OF and IMO-OF groups during the 5 min following the first blood sampling (post-IMO R 0–5) and during the 5 min preceding the second and the third blood sampling (post-IMO R 40–45 and R 85–90, respectively). Blood sampling times were changed with respect to Experiment 1 because IMO is characterized by a slower return of HPA hormones to pre-stress levels as compared to the FS (Márquez et al., 2002). On day 8 (retention), all animals were again exposed to the OF for 15 min and their behavior recorded.

The statistical analysis included one between-subjects factor: GROUP (control-home, IMO-home, IMO-OF). When repeated measures were included in the analysis, the within-subjects factors were SAMPLING TIME (3 levels) for endocrine data or BLOCK (3 levels) for motor activity data.

#### Results

**Figure 4:**
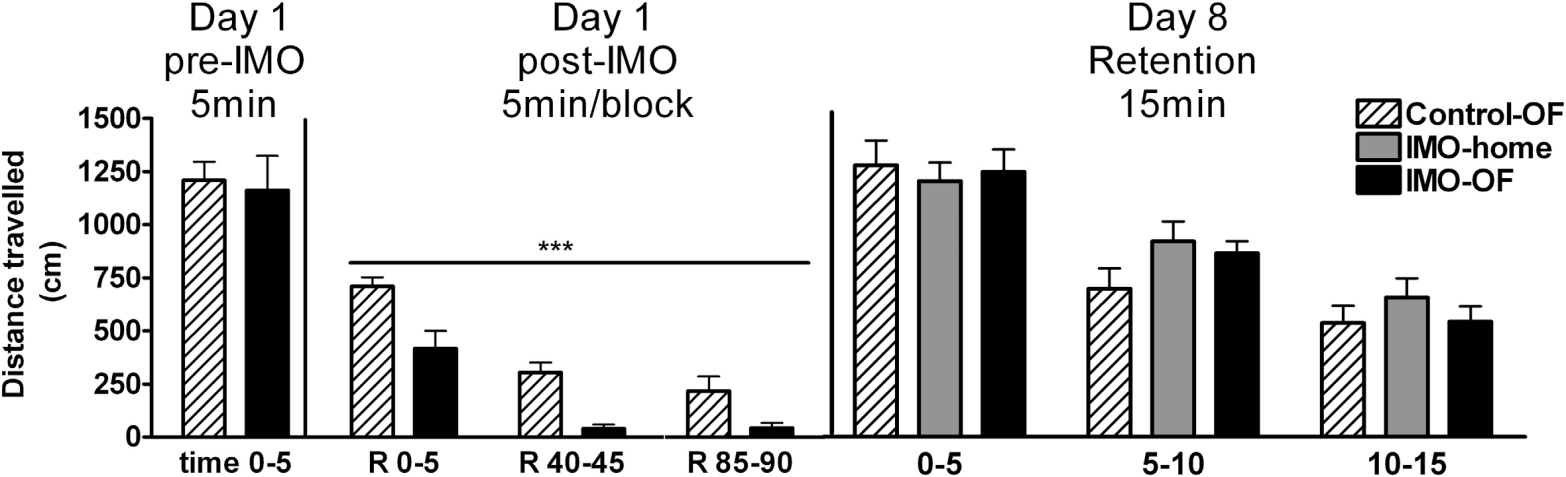
**Distance traveled in an open field (OF) for Experiment 2 on day 1 (pre-IMO and post-IMO period) and day 8 (retention).** Means and SEM are represented. The groups were: control-OF, which were exposed to the OF for 5 min, left undisturbed in their home-cages in the animal room for 25 min and then returned to the OF for an additional 90 min period (R 0–90); IMO-home, which were allowed to explore the OF for 5 min, then exposed to IMO inside the OF for 25 min and finally returned to their home-cages in the animal room; and IMO-OF groups, which were treated as the previous group but were released from the IMO board and maintained in the same OF for an additional post-IMO period of 90 min. On day 8, activity was evaluated in three time blocks of 5 min each. ^***^p < 0.001 vs. control-OF group, regardless of time.

The analysis of baseline activity (pre-IMO time 0–5) revealed no statistically significant differences among groups, whereas statistical analysis of post-IMO activity revealed a significant effect of group (Wald *X*^2^ (1) = 29.84, *p* < 0.001) and block (Wald *X*^2^ (2) = 76.54, *p* < 0.001), with no interaction (Figure 4). These data reflect that exposure to IMO caused a marked inhibition of activity in the OF and that activity was progressively reduced over time in both control and IMO groups due to habituation to the OF.

The analysis of activity of animals in the OF on day 8 (retention, Figure 4) revealed no significant effect of group, but a significant effect of block (Wald *X*^2^ (2) = 120.78, *p* < 0.001), reflecting a progressive decline in activity over the 15 min session.

**Figure 5:**
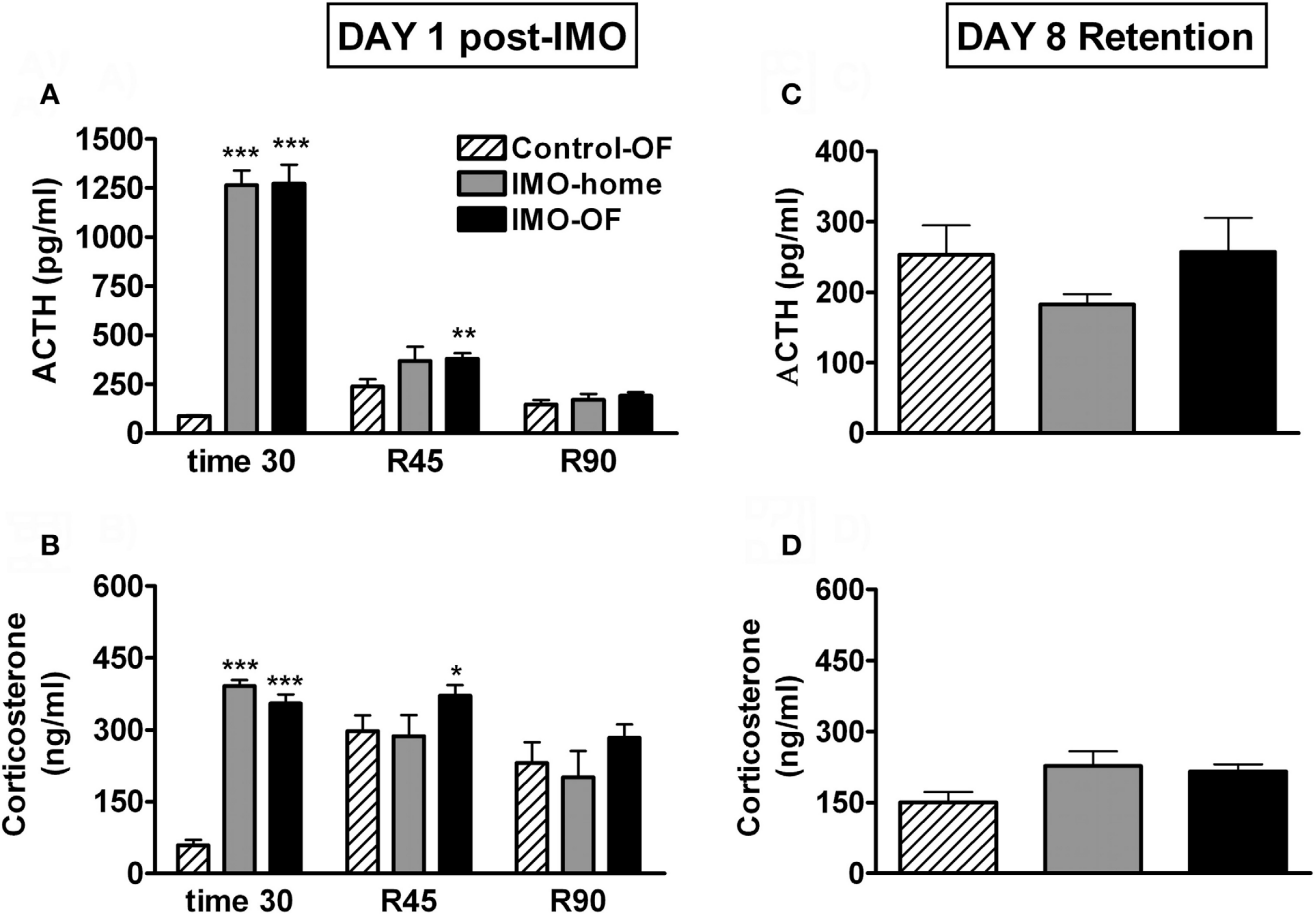
**Plasma ACTH and corticosterone levels for Experiment 2.** Means and SEM are represented. The groups were: control-OF, which were exposed to the OF for 5 min, left undisturbed in their home-cages in the animal room for 25 min and then returned to the OF for an additional 90 min period (R 0–90); IMO-home, which were allowed to explore the OF for 5 min, then exposed to IMO inside the OF for 25 min and finally returned to their home-cages in the animal room; and IMO-OF groups, which were treated as the previous group but were released from the IMO board and maintained in the same OF for an additional post-IMO period of 90 min. Panel **(A** and **B)** show hormone levels on day 1, just after IMO (time 30) and at 45 and 90 min after IMO (R45 and R90). Panel **(C** and **D)** show hormone levels on day 8 (retention). ^*^, ^**^, ^***^: *p* < 0.05, *p* < 0.01 and *p* < 0.001 vs. control-OF group.

Analysis of plasma ACTH levels (Figure 5A) revealed significant effects of group (Wald *X*^2^ (2) = 166.78, *p* < 0.001), sampling time (Wald *X*^2^ (2) = 414.74, *p* < 0.001) and the interaction group *X* sampling time (Wald *X*^2^ (4) = 421.27, *p* < 0.001). Further analysis showed very high levels of ACTH immediately after IMO in the two stressed groups as compared to the control-OF group; during the post-IMO period, the only significant differences among the groups was the higher levels of ACTH of IMO-OF as compared to control-OF group at 45 min post-IMO. Statistical analysis of corticosterone levels (Figure 5B) indicated significant effects of group (Wald *X*^2^ (2) = 29.88, *p* < 0.001), sampling time (Wald *X*^2^ (2) = 22.74, *p* < 0.001) and the interaction group *X* sampling time (Wald *X*^2^ (4) = 65.07, *p* < 0.001). Further analysis revealed the same pattern as ACTH: very high levels of corticosterone immediately after IMO in the two stressed groups as compared to the control-OF group and higher levels of corticosterone in IMO-OF as compared to control-OF group (*p* < 0.01) at 45 min post-IMO. The analysis of HPA response to the OF on day 8 revealed no effect of group for ACTH or corticosterone levels (Figures 5C,D).

Exposure to IMO in a particular environment resulted in a marked activation of the HPA axis. However, contrary to the results obtained after FS in the previous experiment, no obvious endocrine evidence for conditioning was observed in the immediate post-IMO period, considering that IMO-home and IMO-OF groups did not differ in plasma levels of ACTH and corticosterone at any time. IMO-OF rats showed marked hypo-activity in the OF during all the post-IMO period as compared to controls, but this can reflect the unconditioned inhibition of activity caused by exposure to severe stressors (i.e., Reinstein et al., 1984; Pol et al., 1992). More importantly, when animals were exposed again, on day 8, to the OF, no evidence for conditioning was found. Thus, similar levels of activity were observed in all groups, with no evidence for freezing in IMO rats. Moreover, plasma levels of ACTH and corticosterone were similar in all groups, thus supporting the lack of conditioning.

### Experiment 3

The objective of the experiment was to directly study whether the differences in the acquisition of fear conditioning between FS and IMO were related to the different characteristics of the context and/or to a much stronger association to the IMO board, acting as a cue, than to the context in the IMO group. To this end, animals were assigned to three experimental groups (Figure 1C): control, FS and IMO. All animals were individually exposed to the large shock chambers.

We modified the present protocol respect to the preceding ones for three reasons. First, we reduced the time of stress exposure considering that 15 min of acquisition of fear conditioning is enough to develop a very strong fear conditioning as well as to get an appropriate activation of the HPA axis. Second, we did not follow behavior during the post-stress period on day 1 as no relevant additional information was obtained in the preceding experiments. Finally, we exposed the rats to a 15 min testing session to analyze both behavioral and hormonal data.

#### Design

On day 1, all animals were exposed for 5 min to large chambers without receiving FS (pre-shock/IMO time 0–5). After that, the treatment differed in function of the experimental group (Figure 1C): rats from the control group (*n* = 16) were maintained for an additional 15 min period in the chamber without receiving shock. The rats from the FS group (*n* = 8) were exposed for 15 min (shock time 5–20) to 15 shocks in total. The rats from the IMO group (*n* = 24) were immobilized and maintained for 15 min (IMO time 5–20) within the chambers (without FS). After these procedures, all rats were blood sampled and returned to their regular home-cages.

On day 8 (retention), all rats were exposed to the chamber for 15 min to evaluate freezing and motor activity as measures of contextual fear conditioning. In order to know whether the IMO board acted as a cue for the IMO procedure, half of the rats from the control and IMO groups were introduced inside an empty chamber, whereas the other half were exposed to the chamber with the IMO board inside. The IMO board was located in one of the sides of the chamber. A blood sample was taken after the end of the test. Behavior was recorded in the chambers for all groups, in 5 min blocks. The chamber was divided into 3 equal zones (z1, z2, and z3), being z1 the zone where the board was placed and z3 the opposite zone. The time spent in each zone was also evaluated.

The statistical analysis included one between-subjects factor: either STRESS on day 1 (control, FS, and IMO) or GROUP on day 8 (control, FS, IMO, IMO-board). On day 8, both control groups (with or without the board) were treated as a whole because no significant differences between them were detected. When repeated measures were included in the analysis, the within-subjects factor were BLOCK (3 levels) for the motor activity and freezing data or ZONE (3 levels) for time spent in the different sections of the FS chamber.

#### Results

**Figure 6:**
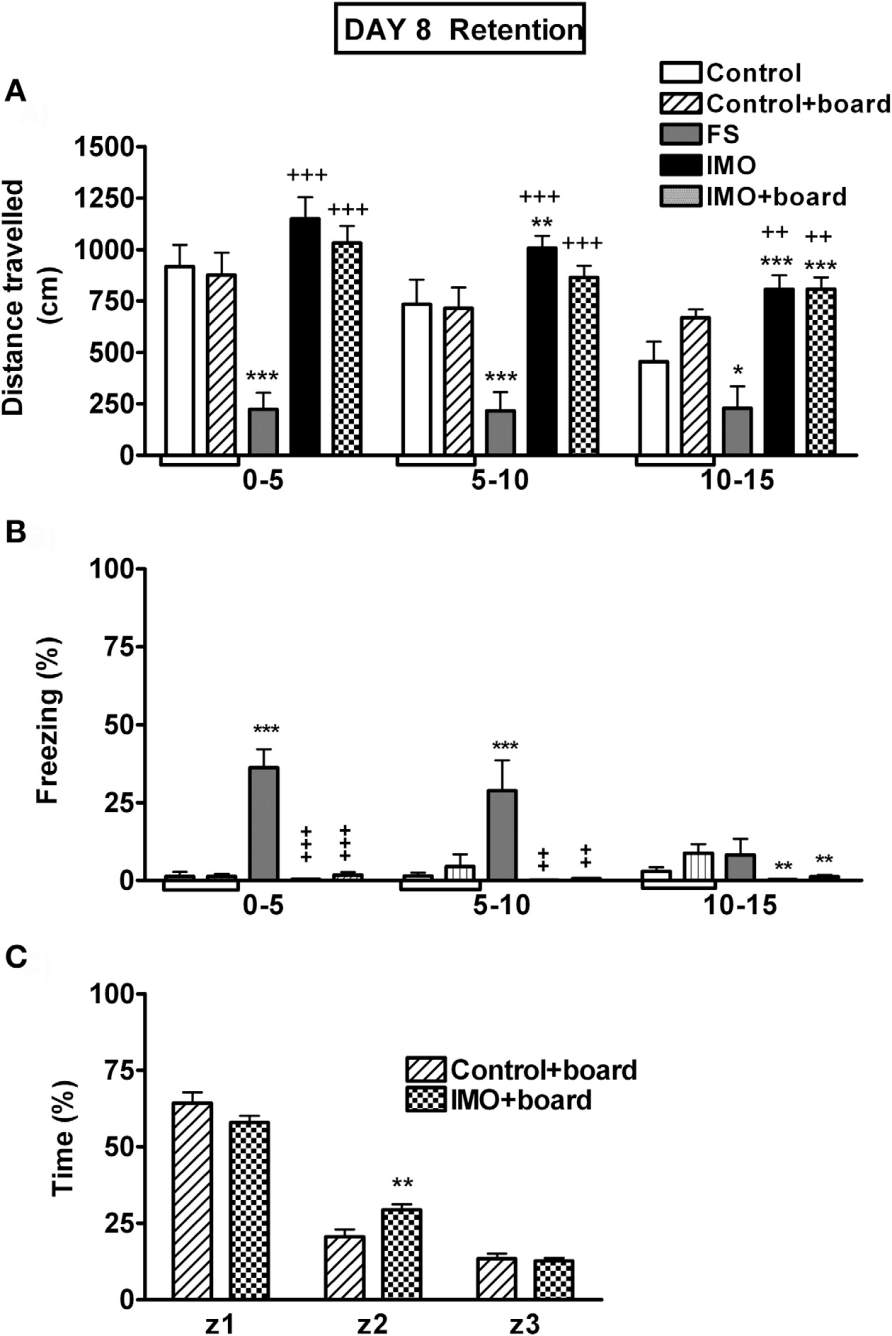
**Behavioral data for Experiment 3 on day 8 (retention).** Means and SEM are represented. The groups were: control, rats exposed for 20 min to the chamber without shocks; FS, rats allowed to explore the chamber for 5 min and then exposed to shocks for 15 min; and IMO, rats allowed to explore the chamber for 5 min and then exposed to IMO inside the chamber for 15 min. On day 8 control and IMO rats were tested without the presence of the board or with the board (control-board and IMO-board groups). Control and control+board groups are presented separately in the graphs but they were pooled for the statistical analysis. Panel **(A)** shows distance traveled during the 15 min exposure to the chamber (5 min blocks). Panel **(B)** shows freezing during the 15 min exposure to the chamber (5 min blocks). Panel **(C)** shows time spent in the area were the board was located (z1), in the intermediate area (z2) or in the opposite area (z3). ^*^, ^**^, ^***^: *p* < 0.05, *p* < 0.01 and *p* < 0.001 vs. control group; ^+^, ^++^, ^+++^: *p* < 0.05, *p* < 0.01 and *p* < 0.001 vs. FS group.

The analysis of baseline activity in the chamber on day 1 revealed no statistically significant differences among groups (data not shown). When animals were exposed again 7 days later to the chamber, without FS or IMO, (Figure 6A), the analysis showed significant effects of group (Wald *X*^2^ (3) = 54.21, *p* < 0.001), block (Wald *X*^2^ (2) = 28.59, *p* < 0.001) and the interaction group *X* block (Wald *X*^2^ (6) = 17.34, *p* < 0.001). The decomposition of interaction indicated that only the FS group showed a significant hypo-activity in the chamber over the three blocks of time in comparison to control animals. IMO groups presented an increase in activity if compared with controls. These data can be explained because the control group had more time to explore the chamber on day 1 (20 min) than the IMO group (5 min).

The analysis of freezing behavior during re-exposure (retention) on day 8 to the conditioned context followed the same pattern as activity (Figure 6B). The factors group (Wald *X*^2^(3) = 42.31, *p* < 0.001) and block (Wald *X*^2^(2) = 12.00, *p* < 0.001) were statistically significant, as well as the interaction group *X* block (Wald *X*^2^ (6) = 28.17, *p* < 0.001). Only the FS group showed increased freezing behavior, reflecting fear conditioning in comparison with control and IMO groups during the first and the second block of time.

At day 8, the time spent in the zone of the chamber were the IMO board was placed was also analyzed. As can be seen in Figure 6C, comparison of the two groups of animals exposed to the chamber with the IMO board present as a cue, revealed that the stress factor was not statistically significant. However, zone (Wald X^2^ (2) = 539.41, *p* < 0.001) and the interaction stress *X* zone (Wald *X*^2^ (2) = 15.47, *p* < 0.01) were statistically significant. The data indicated that there were no differences between control and IMO animals in the time spent in the zone with the IMO board (z1), whereas the IMO group showed slightly higher time in the intermediate zone (z2) than controls.

**Figure 7:**
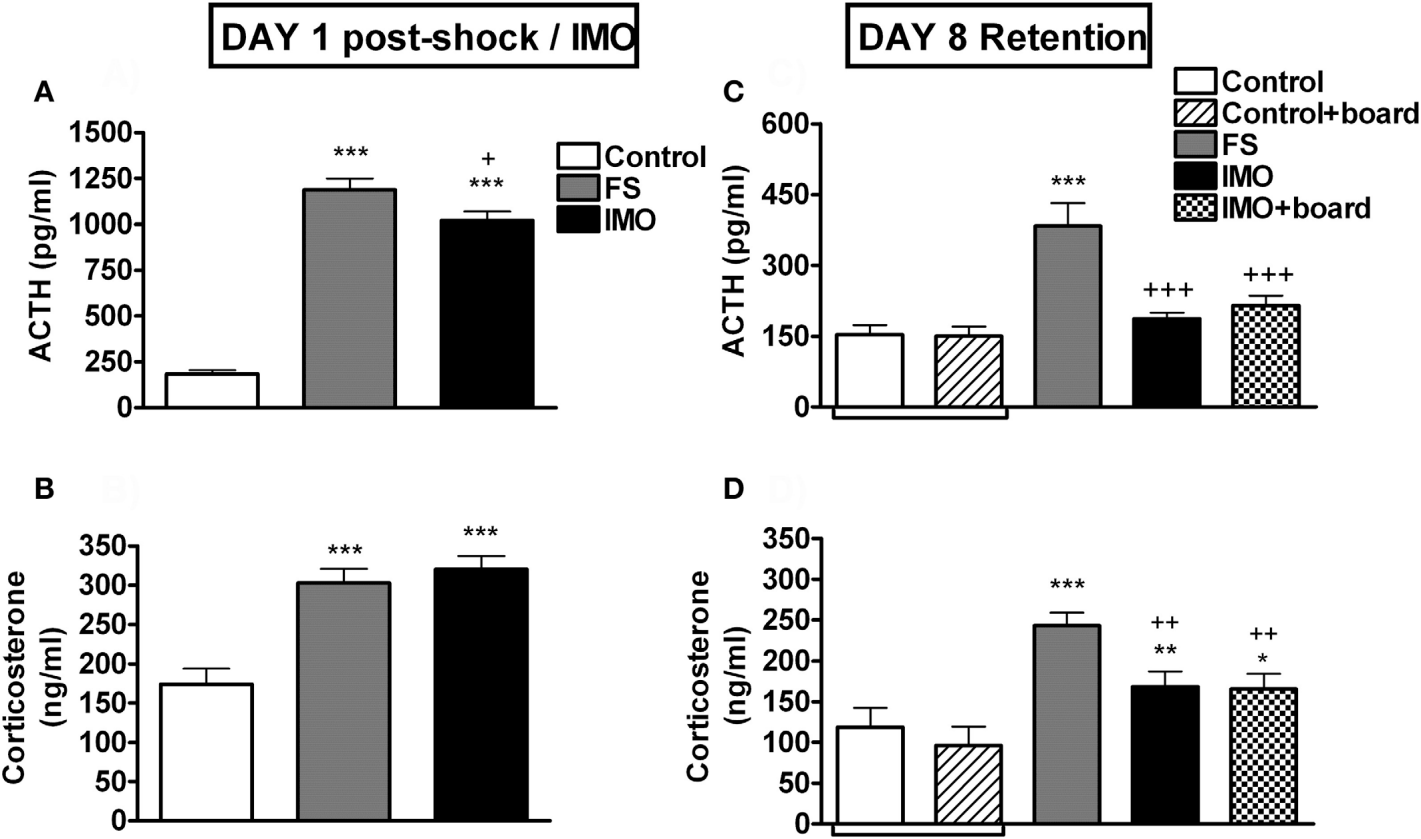
**Plasma ACTH and corticosterone levels for Experiment 3.** Means and SEM are represented. The groups were: control, rats exposed for 20 min to the chamber without shocks; FS, rats allowed to explore the chamber for 5 min and then exposed to shocks for 15 min; and IMO, rats allowed to explore the chamber for 5 min and then exposed to IMO inside the chamber for 15 min. On day 8 control and IMO rats were tested without the presence of the board or with the board (control-board and IMO-board groups). Control and control + board groups are presented separately in the graphs but they were pooled for the statistical analysis. Panel **(A** and **B)** show post-stress hormone levels on day 1. Panel **(C** and **D)** show hormone levels on day 8 (retention). ^*^, ^**^, ^***^: *p* < 0.05, *p* < 0.01 and *p* < 0.001 vs. control group; ^+^, ^++^, ^+++^: *p* < 0.05, *p* < 0.01 and *p* < 0.001 vs. FS group.

The analysis of plasma ACTH on day 1 (Figure 7A) showed a significant effect of stress (Wald *X*^2^ (2) = 238.13, *p* < 0.001). Both FS and IMO groups showed higher levels of ACTH as compared to control groups (*p* < 0.001), and FS also differed from the IMO group (*p* < 0.05). When animals were re-exposed at day 8 to the chamber (Figure 7C), groups differences were again statistically significant (Wald *X*^2^ (3) = 57.06, *p* < 0.001), but in this case the FS group showed higher levels of ACTH as compared to control (*p* < 0.001) and IMO groups (*p* < 0.001), reflecting hormonal fear conditioning. Plasma corticosterone levels on day 1 (Figure 7B) followed the same pattern as ACTH: group effect was statistically significant (Wald *X*^2^ (3) = 38.67, *p* < 0.001), and both stressed groups showed higher levels of corticosterone immediately after stress (*p* < 0.001). The analysis of corticosterone levels after re-exposure to the conditioned context (chamber) at day 8 (Figure 7D) revealed a significant effect of group (Wald *X*^2^ (3) = 28.02, *p* < 0.001): FS group showed the highest levels of corticosterone, differing from controls (*p* < 0.001), IMO (*p* < 0.01) and IMO + board groups (*p* < 0.01). IMO and IMO + board groups showed higher levels of corticosterone than controls (*p* < 0.01, *p* < 0.05 respectively).

As expected, FS and IMO groups showed much higher levels of ACTH and corticosterone than controls immediately after stressors, with FS rats showing slightly higher levels than IMO rats. However, when tested for fear conditioning marked differences emerged between the two groups. Previously shocked rats showed high levels of freezing that progressively decreased over the 15 min period, whereas IMO rats showed very low levels of freezing similar to controls. Similarly, shocked rats showed hypo-activity, whereas IMO rats did not. Importantly, the presence of the IMO board did not modify the behavior of either control or IMO rats, demonstrating that it did not act as a particular cue for fear conditioning. In fact, in the presence of the board, both control and IMO rats spent more time in the area where the board was located than in the opposite area, with no evidence of avoidance in the IMO rats. Supporting behavioral results, FS rats showed higher ACTH and corticosterone responses to the large FS chamber than control and IMO rats. Despite no differences between IMO and controls rats in the ACTH response to the FS chamber, slightly higher levels of corticosterone were observed in IMO and IMO + board rats as compared to controls (*p* < 0.01, *p* < 0.05, respectively).

## Discussion

In the present work we demonstrated that exposure to a single session of FS induced a strong contextual fear conditioning as deduced from the behavioral and endocrine responses, whereas, we were unable to find similar evidence for contextual conditioning after exposure to IMO in a particular environment. The striking contrast between the two stressors regarding contextual fear conditioning was further demonstrated in a last experiment comparing directly the consequences of exposure to the two stressors in the same context. In addition, the last experiment demonstrated that the IMO board did not act as a putative cue. Therefore, fear conditioning appears to develop easier after exposure to certain aversive stimuli than others.

Exposure of animals to single or repeated FS in a specific environment easily results in the development of contextual fear conditioning, but the extent to which exposure to any kind of stressor always results in contextual fear conditioning is unclear. We found in our lab preliminary negative behavioral evidence about the development of contextual fear conditioning to IMO. Therefore, we first decided to demonstrate that, in our conditions, contextual fear conditioning to FS easily develops that should be reflected not only in the standard measure of freezing, but also in the activation of the HPA axis. High levels of freezing were found in the FS chamber group when the rats were returned to the FS chamber immediately after blood sampling and when assessed again at 30 min post-shocks. These data clearly demonstrated that strong contextual fear conditioning developed in FS rats, which was maintained at a high level even 30 min after the termination of FS.

Measurement of plasma levels of ACTH and corticosterone demonstrated that mere exposure to the FS chambers without shocks caused a modest activation of the HPA axis. This is not surprising as the FS chamber constitutes a novel environment for the animals and this consistently elicits activation of the HPA axis (i.e., Márquez et al., 2005). As expected, both FS groups showed a much higher activation of the HPA axis than controls, however, the FS-chamber group showed clearly higher levels of ACTH than the FS-home group. These differences in ACTH between the two FS groups at 30 min post-shock cannot be explained by the slightly higher levels observed in control-chamber as compared to control-home groups. Therefore, it appears that the HPA axis is able to reflect the enhanced fear caused by maintenance of the animals in the environment previously associated to the aversive experience of FS. To our knowledge, the influence of maintaining the rats in the shock environment on the HPA axis has only been previously studied in one single study, with similar results (Gao et al., 2008). It is therefore clear that these results are also in accordance with previous data demonstrating that HPA hormones are quite sensitive to the degree of stress experienced by animals (Armario, 2006) and, more particularly, to fear conditioning (Van de Kar et al., 1991; Campeau et al., 1997; Merino et al., 2000; Muñoz-Abellán et al., 2009; Daviu et al., 2010).

When rats were again exposed 7 days later to the FS context, both FS groups showed the expected high levels of freezing as compared to non-shocked groups. In fact, their levels of freezing were similar as those reported in the FS-chamber group at 30 min post-shock on day 1, indicating that freezing was basically maintained intact over the days, with no evidence for extinction in those rats which were maintained for 30 min in the chamber without additional shocks on day 1.

Once characterized the response to FS we did an analogous design with IMO as the stressor. The results showed that exposure to IMO in a particular environment (OF) was apparently unable to induce contextual fear conditioning, despite the huge activation of the HPA axis elicited by the stressor and its slower post-stress recovery of resting levels. This is a particular characteristic of IMO that is related to their high intensity (Martí et al., 2001; Márquez et al., 2002). In spite of this, no obvious endocrine evidence for conditioning was observed in the immediate post-IMO period in that plasma levels of HPA hormones did not differ in IMO-home and IMO-OF groups during this phase.

Can behavior of rats in the OF during the post-IMO period give us some clues about conditioning?

IMO-OF rats showed marked hypo-activity in the OF during all the post-IMO period as compared to controls. However, such a hypo-activity cannot be considered as a reflection of conditioning as exposure to severe stressors, including IMO, resulted in unconditioned inhibition of activity for some hours after the stressor (i.e., Reinstein et al., 1984; Pol et al., 1992). As freezing was not observed, the results tentatively suggest that exposure to IMO in the OF did not result in the development of contextual fear conditioning. This assumption was supported by the lack of changes in activity and the absence of freezing when IMO animals were exposed again, on day 8, to the OF for 15 min. Importantly, ACTH and corticosterone response during the 15 min re-exposure to the OF were similar in control and IMO rats. As such a period of exposure to the context appears to be optimum for HPA hormones to reflect contextual fear conditioning to FS or cat odor (Muñoz-Abellán et al., 2009; Daviu et al., 2010), the hormonal data add support to the lack of IMO-induced contextual fear conditioning.

The above results may suggest that rats were unable to acquire contextual fear conditioning to IMO. However, it could be argued that in the previous experiments the context was very different with FS and IMO and that the most relevant stimulus for IMO was the presence of the board. To rule out the above explanations, a final experiment was done using the same context (a large chamber) for both FS and IMO. Seven days after the stressors, control, FS, and IMO animals were tested for fear conditioning in the grid box either in the absence of the presence of the IMO board. Both behavioral (freezing and hipo-activity) and hormonal (HPA hormones) data supports that only the previous shocked rats showed strong evidence for contextual fear conditioning. Furthermore, the introduction of the board failed to induce cue-fear conditioning in the IMO-board group. Taken together, the results strongly support the hypothesis that IMO rats were unable to associate stress exposure to either cue or context. It is intriguing that IMO rats tested in the large chamber showed modest but consistent hyperactivity together with a slight increase in the corticosterone (but not ACTH) response to the chamber. Although we do not know the reason for these effects, we can speculate that a high level of arousal, not just fear or anxiety, may explain both enhanced activity and the slightly higher corticosterone response (the discrepancy with ACTH may be explained by a very transient ACTH response not observable at 15 min). In fact, immediate prior exposure to low intensity stressors, which probably promotes arousal, has been found to increase activity/exploration in novel environments (i.e., Katz et al., 1981).

On the basis of prior data (Márquez et al., 2002) and the HPA response to FS and IMO, the two stressors appear to be severe and approximately of the same intensity. Therefore, low severity does not appear to be the reason for the lack of contextual fear conditioning to IMO. In the present experiment IMO rats were allowed to explore the environment before IMO and it is unlikely that they could not learn about the context before experiencing IMO. It is also unlikely that IMO would have induced some kind of amnesic effects about the context. We have recently found that contextual fear conditioning to cat odor is basically unaffected in rats that were allowed to explore an OF containing a cloth impregnated with cat odor before being immobilized and returned in these conditions to the same context for an additional 15 min period (Muñoz-Abellán et al., 2011). These results indicate that IMO is unlikely to interfere with cat odor-induced contextual fear conditioning.

The lack of fear conditioning with IMO may be due to several reasons: the type of US, the type of CS, the procedure involved in the CS-US pairing and the type of measure used to evaluate the CR. As we relied on several different CRs (activity/immobility, avoidance, freezing, and HPA activation), it is unlikely that this was the reason for the differences between FS and IMO. Moreover, by changing the way of transporting the animals or the experimenter and by introducing specific odors in the stress chamber, we have been unable to demonstrate fear conditioning to IMO (unpublished), supporting the incapability of the animals to associate IMO to different types of CS. Another difference between FS and IMO is that the former is a discrete stimulus (with a clear on and off signal), whereas IMO is a continuous stimulus. Therefore, FS rats had more opportunities to associate the context with the aversive stimulus. However, this does not appear to be the main reason for the discrepancies. First, it is well-established in the literature that one single shock is able to induce context fear conditioning (i.e., Radulovic et al., 1998). In fact, we have obtained in rats of the same strain and age as those used in the present experiments strong context fear conditioning with one single-shock (Daviu et al., 2010). Considering that IMO is a severe stressor, association may be observed after a single IMO session, which is not the case. Second, no evidence for contextual fear conditioning was observed when animals were immobilized and released from the board several times in a unique session, maintaining the animals in the context in between (unpublished). Although the latter procedure approached to that of FS, it failed again to find fear conditioning.

As we cannot rule out that fear conditioning to IMO could be established by particular, not yet characterized, CS, the most parsimonious explanation for the present results is that the nature of the US (IMO) somehow makes more difficult the association with a particular CS. This idea fits well with the concept of “preparedness” applied to aversive (fear) conditioning initially proposed by Seligman (1971) and refers to the fact that some CS-US associations are easier to develop because are somehow biologically prepared. That is, animals are not biologically well-prepared to develop contextual fear conditioning to any kind of stressor, but only to a subset of them. The first evidence about biological predisposition to establish CS-US associations was obtained in rats by García and Koelling (1966) demonstrating that gastrointestinal malaise caused by the administration of lithium chloride was associated to the ingestion of a novel taste food (saccharin) but not to an exteroceptive CS such as noise, whereas FS exposure was associated to noise but not to the novel taste. Interestingly, it has been very difficult to observe contextual conditioning using a component of fox odor, trimethylthiazoline (TMT), as US (i.e., Blanchard et al., 2003), despite the fact that TMT is an aversive substance that induce by itself defensive behaviors and activates the HPA axis (Morrow et al., 2000). Within this framework, it appears that the critical role of glucocorticoids to strengthening CS-US associations would be dependent on the pre-existence of neuronal circuits allowing the convergence of information concerning CS and US in the basolateral complex of the amygdala.

The present results not only demonstrate that induction of contextual fear conditioning using standard procedures may be dependent on the type of stressor, but they also have implications regarding the evaluation of putative animal models of PTSD. Exposure to certain stressors, including predator odor and FS, has been reported to induce long-lasting (days to weeks) changes in activity in novel environment and/or anxiety-like behavior as measured in the elevated plus-maze (see Introduction) and this has been considered to be important for their characterization as putative animal models of PTSD. In contrast, IMO is a severe stressor from a physiological point of view, but no changes in anxiety-like behavior as evaluated in the EPM or activity in novel environments is usually observed after the first week post-IMO (Belda et al., 2004, 2008). Similarly, reduced social interaction caused by a tail-shock procedure used in the standard learned helplessness paradigm dissipated on 3 days (Maier, 1984) and the effects of the procedure on the EPM are not consistent even during the first 24 h (Grahn et al., 1995). However, IMO can induce long-lasting endocrine and behavioral sensitization (in terms of anxiety) to further stressors (Belda et al., 2008) as well as long-lasting impairment of spatial memory in the Morris water maze (Andero et al., 2012) and fear extinction (Andero et al., 2011), changes both that mimics those reported in PTSD patients (McNally, 1998; Yehuda and LeDoux, 2007; Moore, 2009).

We have suggested that, at least, some of the long-lasting changes in activity or anxiety-like behavior observed after a single exposure to some stressors may be related to their proneness to induce contextual fear conditioning rather than with their traumatic nature (Armario et al., 2008). In fact, we have recently demonstrated that hypo-activity in novel environment is observed even 12 days after a single exposure to FS and this effect disappeared with a procedure that impeded the induction of contextual fear conditioning (Daviu et al., 2010). Although some authors have reported enhanced startle response for 7–10 days after one or three sessions of tail-shock (i.e., Servatius et al., 1995; Manion et al., 2007), the effects were not observed during the first 3 days, suggesting some incubation process, probably involved non-associative memory. Persistence of non-associative effects of severe stressors is likely to differ between individuals (species, strains, environmental conditions). It would be important to directly demonstrate the direct relationship between the establishment of contextual fear memory and the persistence of long-lasting changes in anxiety-like behavior. If such a relationship holds true, it could question some putative animal models of PTSD.

In conclusion, the present results indicate that acquisition of contextual fear conditioning is extremely easy with FS as aversive stimulus, whereas appears to be extremely difficult to be acquired using a stressor such as IMO, which is of a similar severity as high intensity FS. A good correlation was observed between behavioral signs of fear conditioning and the activation of the HPA axis, thus confirming previous reports demonstrating that plasma levels of ACTH and corticosterone are able to specifically reflect fear conditioning (see Armario et al., 2012 for further discussion). If some of the long-lasting behavioral effects of stress when tested in novel environments are dependent on the acquisition of contextual fear conditioning, the difficulties of animals to acquire IMO fear conditioning can explain the failure to find some long-lasting effects when free behavior in novel environments is tested. The present data introduce some caveats regarding development of animal models of PTSD.

### Conflict of interest statement

The authors declare that the research was conducted in the absence of any commercial or financial relationships that could be construed as a potential conflict of interest.
